# (3,4-Dimeth­oxy­phen­yl)(4-fluoro­phen­yl)methanone

**DOI:** 10.1107/S1600536810021008

**Published:** 2010-06-05

**Authors:** Quanjian Lv, Jianling Wang

**Affiliations:** a Department of Quality Detection and Management, Zhengzhou College of Animal Husbandry Engineering, Zhengzhou 450011, People’s Republic of China

## Abstract

In the title compound, C_15_H_13_FO_3_, the dihedral angle between the two aromatic rings is 52.78 (8)°. In the crystal, inter­molecular C—H⋯O hydrogen bonds link mol­ecules into chains running parallel to the *c* axis.

## Related literature

For applications of benzophenone and its derivatives, see: Riechers *et al.* (1996 ▶); Khanum *et al.* (2009 ▶); Schlecht *et al.* (2008 ▶).
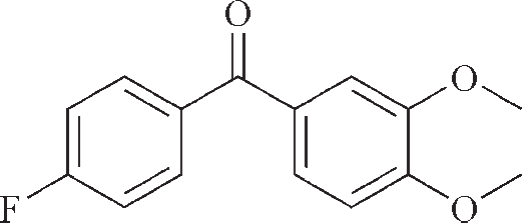


## Experimental

### 

#### Crystal data

C_15_H_13_FO_3_
*M* *_r_* = 260.25
Monoclinic, 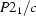

*a* = 10.8926 (9) Å
*b* = 11.3632 (11) Å
*c* = 10.8369 (10) Å
β = 108.285 (1)°
*V* = 1273.6 (2) Å^3^
*Z* = 4
Mo *K*α radiation
μ = 0.10 mm^−1^
*T* = 298 K
0.48 × 0.38 × 0.15 mm

#### Data collection

Bruker SMART CCD area-detector diffractometer
Absorption correction: multi-scan (*SADABS*; Sheldrick, 1996 ▶) *T*_min_ = 0.952, *T*_max_ = 0.985
6183 measured reflections
2227 independent reflections
1391 reflections with *I* > 2σ(*I*)
*R*_int_ = 0.083

#### Refinement

*R*[*F* ^2^ > 2σ(*F* ^2^)] = 0.056
*wR*(*F* ^2^) = 0.173
*S* = 1.00
2227 reflections
174 parameters
H-atom parameters constrained
Δρ_max_ = 0.20 e Å^−3^
Δρ_min_ = −0.32 e Å^−3^

### 

Data collection: *SMART* (Siemens, 1996 ▶); cell refinement: *SAINT* (Siemens, 1996 ▶); data reduction: *SAINT*; program(s) used to solve structure: *SHELXS97* (Sheldrick, 2008 ▶); program(s) used to refine structure: *SHELXL97* (Sheldrick, 2008 ▶); molecular graphics: *SHELXTL* (Sheldrick, 2008 ▶); software used to prepare material for publication: *SHELXTL*.

## Supplementary Material

Crystal structure: contains datablocks I, global. DOI: 10.1107/S1600536810021008/rz2456sup1.cif

Structure factors: contains datablocks I. DOI: 10.1107/S1600536810021008/rz2456Isup2.hkl

Additional supplementary materials:  crystallographic information; 3D view; checkCIF report

## Figures and Tables

**Table 1:** Hydrogen-bond geometry (Å, °)

*D*—H⋯*A*	*D*—H	H⋯*A*	*D*⋯*A*	*D*—H⋯*A*
C7—H7⋯O1^i^	0.93	2.58	3.343 (3)	139

Symmetry code: (i) 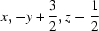.

**Fig. 1.**
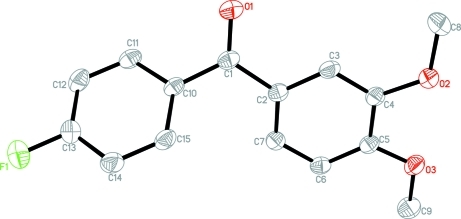
The molecular structure of the compound, with atom labels and 50% probability displacement ellipsoids. Hydrogen atoms are omitted.

**Fig. 2.**
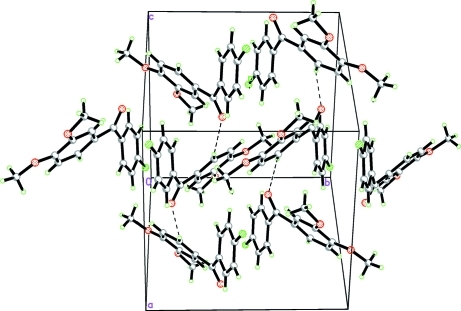
Crystal packing of the title compound. Intermolecular hydrogen bonds are shown as dashed lines.

## supplementary crystallographic information

### Comment

Benzophenone derivatives are an important class of compounds having a broad spectrum of applications in the chemical and biochemical fields (Riechers *et al.*, 1996; Khanum *et al.*, 2009), and are widely used as UV-screens to protect industrial products from light induced damage (Schlecht *et al.*, 2008). In order to develop new applications for benzophenone and its derivatives, structural modifications of benzophenone have been extensively investigated. As a contribution to this field, we report here the crystal structure of the title compound.

The molecular structure of title compound is shown in Fig. 1. The dihedral angle formed by the benzene rings 52.78 (8)°. In the crystal packing (Fig. 2), intermolecular C—H···O hydrogen bonds (Table 1) link molecules into chains running parallel to the *c* axis.

### Experimental

To a solution of 1,2-dimethoxybenzene (138 g, 1.00 mol) in dichloromethane (2.00 l), AlCl_3_ (199 g, 1.50 mol) was added. Then 4-fluorobenzoyl chloride (158 g, 2.00 mol) was added. The mixture was stirred at room temperature for 2 h, followed by filtration and purification by crystallization from ethyl acetate, giving the title compound as a colourless crystalline solid.

### Refinement

All H atoms were placed geometrically and treated as riding on their parent atoms, with C—H = 0.93-0.96 Å and *U*_iso_(H) = 1.2*U*_eq_(C) or 1.5*U*_eq_(C) for methyl H atoms.

### Crystal data

**Table:**

C_15_H_13_FO_3_	*F*(000) = 544
*M**_r_* = 260.25	*D*_x_ = 1.357 Mg m^−^^3^
Monoclinic, *P*2_1_/*c*	Mo *K*α radiation, λ = 0.71073 Å
Hall symbol: -P 2ybc	Cell parameters from 1872 reflections
*a* = 10.8926 (9) Å	θ = 2.7–26.1°
*b* = 11.3632 (11) Å	µ = 0.10 mm^−^^1^
*c* = 10.8369 (10) Å	*T* = 298 K
β = 108.285 (1)°	Needle, colourless
*V* = 1273.6 (2) Å^3^	0.48 × 0.38 × 0.15 mm
*Z* = 4	

### Data collection

**Table:**

Bruker SMART CCD area-detector diffractometer	2227 independent reflections
Radiation source: fine-focus sealed tube	1391 reflections with *I* > 2σ(*I*)
graphite	*R*_int_ = 0.083
phi and ω scans	θ_max_ = 25.0°, θ_min_ = 2.0°
Absorption correction: multi-scan (*SADABS*; Sheldrick, 1996)	*h* = −7→12
*T*_min_ = 0.952, *T*_max_ = 0.985	*k* = −13→13
6183 measured reflections	*l* = −12→12

### Refinement

**Table:**

Refinement on *F*^2^	Primary atom site location: structure-invariant direct methods
Least-squares matrix: full	Secondary atom site location: difference Fourier map
*R*[*F*^2^ > 2σ(*F*^2^)] = 0.056	Hydrogen site location: inferred from neighbouring sites
*wR*(*F*^2^) = 0.173	H-atom parameters constrained
*S* = 1.00	*w* = 1/[σ^2^(*F*_o_^2^) + (0.0915*P*)^2^] where *P* = (*F*_o_^2^ + 2*F*_c_^2^)/3
2227 reflections	(Δ/σ)_max_ < 0.001
174 parameters	Δρ_max_ = 0.20 e Å^−^^3^
0 restraints	Δρ_min_ = −0.32 e Å^−^^3^

### Special details

**Table:**

Geometry. All e.s.d.'s (except the e.s.d. in the dihedral angle between two l.s. planes) are estimated using the full covariance matrix. The cell e.s.d.'s are taken into account individually in the estimation of e.s.d.'s in distances, angles and torsion angles; correlations between e.s.d.'s in cell parameters are only used when they are defined by crystal symmetry. An approximate (isotropic) treatment of cell e.s.d.'s is used for estimating e.s.d.'s involving l.s. planes.
Refinement. Refinement of *F*^2^ against ALL reflections. The weighted *R*-factor *wR* and goodness of fit *S* are based on *F*^2^, conventional *R*-factors *R* are based on *F*, with *F* set to zero for negative *F*^2^. The threshold expression of *F*^2^ > σ(*F*^2^) is used only for calculating *R*-factors(gt) *etc*. and is not relevant to the choice of reflections for refinement. *R*-factors based on *F*^2^ are statistically about twice as large as those based on *F*, and *R*- factors based on ALL data will be even larger.

### Fractional atomic coordinates and isotropic or equivalent isotropic displacement parameters (Å^2^)

**Table:**

	*x*	*y*	*z*	*U*_iso_*/*U*_eq_	
F1	0.13858 (16)	0.52452 (18)	−0.33034 (17)	0.0864 (7)	
O1	0.46324 (18)	0.62209 (18)	0.24672 (19)	0.0638 (6)	
O2	0.88706 (15)	0.85244 (17)	0.40525 (17)	0.0555 (6)	
O3	0.86314 (15)	1.01546 (15)	0.23481 (17)	0.0512 (5)	
C1	0.4782 (2)	0.6672 (2)	0.1495 (3)	0.0433 (6)	
C2	0.5825 (2)	0.7552 (2)	0.1622 (2)	0.0384 (6)	
C3	0.6868 (2)	0.7580 (2)	0.2777 (2)	0.0420 (6)	
H3	0.6928	0.7015	0.3414	0.050*	
C4	0.7801 (2)	0.8432 (2)	0.2976 (2)	0.0405 (6)	
C5	0.7682 (2)	0.9316 (2)	0.2037 (2)	0.0392 (6)	
C6	0.6666 (2)	0.9291 (2)	0.0898 (2)	0.0416 (6)	
H6	0.6597	0.9865	0.0269	0.050*	
C7	0.5739 (2)	0.8405 (2)	0.0688 (2)	0.0399 (6)	
H7	0.5057	0.8387	−0.0086	0.048*	
C8	0.8996 (3)	0.7711 (3)	0.5078 (3)	0.0682 (9)	
H8A	0.8272	0.7790	0.5397	0.102*	
H8B	0.9782	0.7867	0.5768	0.102*	
H8C	0.9020	0.6925	0.4761	0.102*	
C9	0.8510 (3)	1.1108 (3)	0.1468 (3)	0.0664 (9)	
H9A	0.8506	1.0811	0.0637	0.100*	
H9B	0.9225	1.1637	0.1798	0.100*	
H9C	0.7715	1.1519	0.1374	0.100*	
C10	0.3889 (2)	0.6332 (2)	0.0193 (2)	0.0396 (6)	
C11	0.2630 (2)	0.6010 (2)	0.0084 (3)	0.0475 (7)	
H11	0.2355	0.6042	0.0813	0.057*	
C12	0.1782 (3)	0.5644 (2)	−0.1084 (3)	0.0545 (7)	
H12	0.0938	0.5431	−0.1154	0.065*	
C13	0.2215 (3)	0.5604 (3)	−0.2141 (3)	0.0554 (8)	
C14	0.3456 (3)	0.5881 (3)	−0.2083 (3)	0.0559 (8)	
H14	0.3728	0.5816	−0.2812	0.067*	
C15	0.4290 (2)	0.6260 (2)	−0.0911 (3)	0.0462 (7)	
H15	0.5131	0.6470	−0.0854	0.055*	

### Atomic displacement parameters (Å^2^)

**Table:**

	*U*^11^	*U*^22^	*U*^33^	*U*^12^	*U*^13^	*U*^23^
F1	0.0788 (12)	0.1100 (15)	0.0628 (12)	−0.0321 (12)	0.0113 (9)	−0.0168 (10)
O1	0.0677 (13)	0.0799 (14)	0.0502 (12)	−0.0215 (11)	0.0274 (10)	0.0033 (10)
O2	0.0450 (11)	0.0706 (13)	0.0473 (11)	−0.0113 (9)	0.0094 (9)	0.0127 (9)
O3	0.0463 (11)	0.0513 (11)	0.0573 (12)	−0.0076 (9)	0.0183 (8)	0.0060 (9)
C1	0.0400 (14)	0.0476 (15)	0.0491 (16)	0.0021 (12)	0.0239 (12)	0.0029 (12)
C2	0.0338 (13)	0.0456 (14)	0.0422 (14)	0.0041 (11)	0.0210 (10)	−0.0028 (11)
C3	0.0398 (14)	0.0480 (15)	0.0446 (15)	0.0002 (12)	0.0223 (11)	0.0055 (12)
C4	0.0317 (13)	0.0504 (15)	0.0427 (15)	0.0043 (12)	0.0165 (11)	0.0016 (12)
C5	0.0366 (13)	0.0412 (14)	0.0457 (15)	0.0032 (12)	0.0215 (11)	−0.0022 (11)
C6	0.0440 (14)	0.0427 (14)	0.0433 (14)	0.0050 (12)	0.0210 (11)	0.0036 (11)
C7	0.0353 (13)	0.0463 (14)	0.0402 (14)	0.0060 (12)	0.0148 (10)	−0.0010 (11)
C8	0.0643 (19)	0.080 (2)	0.0492 (17)	−0.0084 (17)	0.0021 (14)	0.0185 (16)
C9	0.076 (2)	0.0543 (17)	0.069 (2)	−0.0170 (16)	0.0224 (16)	0.0105 (15)
C10	0.0358 (13)	0.0402 (14)	0.0490 (16)	−0.0007 (11)	0.0225 (11)	0.0006 (11)
C11	0.0426 (14)	0.0522 (16)	0.0568 (17)	−0.0010 (13)	0.0286 (12)	0.0019 (13)
C12	0.0389 (14)	0.0595 (18)	0.0688 (19)	−0.0084 (14)	0.0223 (13)	−0.0028 (15)
C13	0.0566 (17)	0.0554 (18)	0.0515 (17)	−0.0119 (15)	0.0132 (13)	−0.0072 (14)
C14	0.0630 (18)	0.0610 (18)	0.0541 (18)	−0.0105 (15)	0.0332 (14)	−0.0102 (14)
C15	0.0396 (14)	0.0513 (15)	0.0560 (17)	−0.0068 (13)	0.0267 (12)	−0.0057 (13)

### Geometric parameters (Å, °)

**Table:**

F1—C13	1.362 (3)	C8—H8A	0.9600
O1—C1	1.227 (3)	C8—H8B	0.9600
O2—C4	1.370 (3)	C8—H8C	0.9600
O2—C8	1.419 (3)	C9—H9A	0.9600
O3—C5	1.368 (3)	C9—H9B	0.9600
O3—C9	1.422 (3)	C9—H9C	0.9600
C1—C2	1.488 (3)	C10—C11	1.388 (3)
C1—C10	1.493 (3)	C10—C15	1.399 (3)
C2—C7	1.383 (3)	C11—C12	1.377 (4)
C2—C3	1.402 (3)	C11—H11	0.9300
C3—C4	1.370 (3)	C12—C13	1.370 (4)
C3—H3	0.9300	C12—H12	0.9300
C4—C5	1.407 (4)	C13—C14	1.370 (4)
C5—C6	1.376 (3)	C14—C15	1.378 (4)
C6—C7	1.393 (3)	C14—H14	0.9300
C6—H6	0.9300	C15—H15	0.9300
C7—H7	0.9300		
			
C4—O2—C8	117.6 (2)	H8A—C8—H8C	109.5
C5—O3—C9	117.5 (2)	H8B—C8—H8C	109.5
O1—C1—C2	120.3 (2)	O3—C9—H9A	109.5
O1—C1—C10	118.7 (2)	O3—C9—H9B	109.5
C2—C1—C10	120.9 (2)	H9A—C9—H9B	109.5
C7—C2—C3	119.2 (2)	O3—C9—H9C	109.5
C7—C2—C1	121.9 (2)	H9A—C9—H9C	109.5
C3—C2—C1	118.6 (2)	H9B—C9—H9C	109.5
C4—C3—C2	120.6 (2)	C11—C10—C15	118.5 (2)
C4—C3—H3	119.7	C11—C10—C1	118.8 (2)
C2—C3—H3	119.7	C15—C10—C1	122.6 (2)
C3—C4—O2	125.4 (2)	C12—C11—C10	121.2 (2)
C3—C4—C5	119.7 (2)	C12—C11—H11	119.4
O2—C4—C5	114.9 (2)	C10—C11—H11	119.4
O3—C5—C6	124.7 (2)	C13—C12—C11	118.1 (2)
O3—C5—C4	115.4 (2)	C13—C12—H12	121.0
C6—C5—C4	119.9 (2)	C11—C12—H12	121.0
C5—C6—C7	120.0 (2)	F1—C13—C12	118.7 (2)
C5—C6—H6	120.0	F1—C13—C14	118.0 (3)
C7—C6—H6	120.0	C12—C13—C14	123.2 (3)
C2—C7—C6	120.5 (2)	C13—C14—C15	118.0 (3)
C2—C7—H7	119.8	C13—C14—H14	121.0
C6—C7—H7	119.8	C15—C14—H14	121.0
O2—C8—H8A	109.5	C14—C15—C10	120.9 (2)
O2—C8—H8B	109.5	C14—C15—H15	119.5
H8A—C8—H8B	109.5	C10—C15—H15	119.5
O2—C8—H8C	109.5		
			
O1—C1—C2—C7	−153.7 (3)	C3—C2—C7—C6	−0.9 (3)
C10—C1—C2—C7	25.5 (3)	C1—C2—C7—C6	173.2 (2)
O1—C1—C2—C3	20.4 (3)	C5—C6—C7—C2	0.5 (3)
C10—C1—C2—C3	−160.4 (2)	O1—C1—C10—C11	30.4 (3)
C7—C2—C3—C4	−0.7 (4)	C2—C1—C10—C11	−148.8 (2)
C1—C2—C3—C4	−175.1 (2)	O1—C1—C10—C15	−145.9 (3)
C2—C3—C4—O2	−178.5 (2)	C2—C1—C10—C15	34.9 (3)
C2—C3—C4—C5	2.7 (4)	C15—C10—C11—C12	−1.0 (4)
C8—O2—C4—C3	−3.3 (4)	C1—C10—C11—C12	−177.4 (2)
C8—O2—C4—C5	175.4 (2)	C10—C11—C12—C13	0.2 (4)
C9—O3—C5—C6	4.3 (3)	C11—C12—C13—F1	−179.9 (2)
C9—O3—C5—C4	−176.1 (2)	C11—C12—C13—C14	1.6 (5)
C3—C4—C5—O3	177.30 (19)	F1—C13—C14—C15	179.1 (2)
O2—C4—C5—O3	−1.6 (3)	C12—C13—C14—C15	−2.4 (5)
C3—C4—C5—C6	−3.1 (4)	C13—C14—C15—C10	1.5 (4)
O2—C4—C5—C6	178.1 (2)	C11—C10—C15—C14	0.2 (4)
O3—C5—C6—C7	−179.0 (2)	C1—C10—C15—C14	176.5 (2)
C4—C5—C6—C7	1.4 (4)		

### Hydrogen-bond geometry (Å, °)

**Table:**

*D*—H···*A*	*D*—H	H···*A*	*D*···*A*	*D*—H···*A*
C7—H7···O1^i^	0.93	2.58	3.343 (3)	139

Symmetry codes: (i) *x*, −*y*+3/2, *z*−1/2.

## References

[bb1] Khanum, S. A., Girish, V., Suparshwa, S. S., Khanum, N. F. (2009). *Bioorg. Med. Chem. Lett.* **19**, 1887–1891. 10.1016/j.bmcl.2009.02.070 19272777

[bb2] Riechers, H., Albrecht, H.-P. & Amberg, W. (1996). *J. Med. Chem.* **39**, 2123–2128.. 10.1021/jm960274q 8667356

[bb3] Schlecht, C., Klammer, H., Frauendorf, H., Wuttke, W. & Jarry, H. (2008). *Toxicology*, **245**, 11–17. 10.1016/j.tox.2007.12.015 18242814

[bb4] Sheldrick, G. M. (1996). *SADABS* University of Göttingen, Germany.

[bb5] Sheldrick, G. M. (2008). *Acta Cryst.* A**64**, 112–122. 10.1107/S0108767307043930 18156677

[bb6] Siemens (1996). *SMART* and *SAINT* Siemens Analytical X-ray Instruments Inc., Madison, Wisconsin, USA.

